# “How will we cope?” Couples With Intellectual Disability Where One Partner Has a Diagnosis of Dementia

**DOI:** 10.1093/geront/gnae030

**Published:** 2024-03-20

**Authors:** Karen Watchman, Paula Jacobs, Louise Boustead, Andrew Doyle, Lynn Doyle, Jan Murdoch, Jill Carson, Louise Hoyle, Heather Wilkinson

**Affiliations:** Faculty of Health Sciences and Sport, University of Stirling, Stirling, UK; Faculty of Health Sciences and Sport, University of Stirling, Stirling, UK; School of Health in Social Science, University of Edinburgh, Edinburgh, UK; Independent Research Consultant with Intellectual Disability, Dumfries and Galloway, UK; Independent Research Consultant with Intellectual Disability, Dumfries and Galloway, UK; Independent Research Consultant with Intellectual Disability, Dumfries and Galloway, UK; Key and Community Lifestyles, Glasgow, UK; Alzheimer Scotland, Glasgow, UK; Faculty of Health Sciences and Sport, University of Stirling, Stirling, UK; School of Health in Social Science, University of Edinburgh, Edinburgh, UK

**Keywords:** Aging, Coproduction, Down syndrome, Relationships, Social care

## Abstract

**Background and Objectives:**

People with intellectual disability are at increased risk of dementia at an earlier age. This is the first study to explore experiences of couples with an intellectual disability when one partner has dementia.

**Research Design and Methods:**

Four people with intellectual disability whose partner had dementia and one partner who had both an intellectual disability and dementia took part in narrative life story interviews. One of the interviews was conducted as a couple giving direct perspectives from 4 couples overall. Additionally, 13 semistructured interviews were conducted with 9 social care professionals and 4 family members. This provided perspectives of the relationships of a further 4 couples, which collectively led to data on 8 couples.

**Results:**

The emotional impact of a dementia diagnosis, planning for the future, and fear of separation was noted by couples with intellectual disability. Partners took on caring roles thus challenging views of being solely care-receivers. Families spoke of commitment and longevity in relationships, whilst social care staff highlighted how their own information needs changed recognizing the importance of intellectual disability and dementia-specific knowledge.

**Discussion and Implications:**

Couples with intellectual disability continue to enjoy intimate relationships into later life and will face common conditions in older age including dementia. Those who provide support need to ensure that they are sensitive to the previous experience and life story of each couple and have specific knowledge of how dementia can affect people with intellectual disability.

## Background and Objectives

The concept for this research came from one of the coauthors, Andrew, a married man with intellectual disability who had dementia. Demonstrating a coproduced approach from the outset, he provided the impetus for the grant application and, along with two others with intellectual disability informed all stages of the work as part of a research advisory group.

People with intellectual disability are at increased risk of dementia at a younger age, 13.1% of those aged 60 and older and 19.3% of those aged 65 and older compared to 1% of people aged 60–65 without intellectual disability and 13% of 80–85 year olds (British Psychological Society, 2015). This figure is even higher in people with Down syndrome; of around 47,000 people with Down syndrome in the UK, and 400,000 in the United States, two in three (67%) will have dementia by their mid-60s (Alzheimer’s Society, 2023; National Down Syndrome Society, 2023). Yet, people with intellectual disability and dementia remain largely overlooked in dementia research with few studies including this population as participants to explore their perspectives (Jacobs, Watchman, Wilkinson, & Hoyle, 2023). This has resulted in a limited evidence base from which to understand how best to support people. Following the closure of large-scale institutions in the UK for people with intellectual disability, most of which took place from the mid-1980s to the mid-2000s, the majority of people with intellectual disability have been supported in the community, either in their own home or in small group living arrangements.

A scoping review and a systematic review conducted by the research team (Jacobs, Watchman, Wilkinson, & Hoyle, 2023) established that no studies to date have looked at experiences of couples who have an intellectual disability when one partner has dementia. Consequently, it is not known what coping mechanisms are developed or how couples with intellectual disability wish to be supported. The United National Convention on the Rights of Persons with Disabilities (United Nations, 2007) recognizes and advocates for the right of people with intellectual disability to enjoy relationships, get married, and have a family. People with intellectual disability are now living longer and it remains important to celebrate relationships, while also recognizing that many people continue to face barriers to intimate relationships (McCarthy et al., 2021). Additionally, older couples are likely to experience socioeconomic disadvantages in relation to financial resources, not owning their homes and having smaller informal support networks compared to couples without intellectual disability (Power & Bartlett, 2019). Conversely, the importance of supporting relationships for people’s well-being, sense of identity, and belonging has been recognized in research for couples without intellectual disability affected by dementia (Wadham et al., 2016). The reviews identified the importance of emotional support after a diagnosis to help process information about dementia and to plan for the future (Holdsworth & McCabe, 2018).

Coauthor Andrew’s experience after his dementia diagnosis highlighted the lack of an evidence base from which to develop practice that supports couples with intellectual disability. This, and the knowledge that it will continue to be an issue for people with intellectual disability as they enjoy both longer life and long-term relationships, were the drivers for this research. The study was named Stand by Me by the research advisory group as this was the song played during the first dance at Andrew’s wedding to his wife Lynn.

Whilst the life stories of the eight couples are presented elsewhere https://www.scld.org.uk/stand-by-me/, this article focuses on the single research question: to understand experiences of couples with an intellectual disability from multiple perspectives. It discusses the approach taken to include people with intellectual disability in research including the seldom-heard voice of a participant with intellectual disability and dementia.

## Research Design and Methods

People with intellectual disability are often reduced to their diagnoses; life stories can challenge stereotypical views by showing the richness of people’s everyday lives and emphasizing personhood (Ledger et al., 2022). This was an applied study drawing on principles of coproduction in order to develop understanding of relationships and care. We combined multiple case study methodology (Miles & Huberman, 1994) and narrative life story research (Ledger et al., 2022), with one case referring to the story of one couple, which resulted in eight cases in total. A case study approach allowed for an exploration of each couple’s relationship and the impact of dementia from multiple perspectives. This enabled us to detail, where possible, the experiences of the couple or one partner, as well as their close support networks of families and professionals. Narrative life story interviews enabled couples to guide the researcher through their relationship highlighting key people in their lives, important spaces and localities, and key events. This approach has a longstanding tradition in inclusive intellectual disability research (Grove, 2012) as it captures stories of people whose lives are largely undocumented.

### Advisory Group

Continuing an inclusive research approach, a research advisory group was formed at the start of the study to guide the development of the research. This group included three people with intellectual disability (all coauthors), one of whom had dementia and two who were a married couple. Additionally, the group included a representative of a not-for-profit intellectual disability support provider, Key and Community Lifestyles, and from Scotland’s main dementia charity, Alzheimer Scotland, who provide support and information for people with dementia, their carers and families, and campaign for the rights of people affected by dementia. Working closely with the research advisory group enabled us to draw on the expertise of those with lived experience to fully reflect and address needs and concerns as well as ensuring representation from both intellectual disability and dementia service provider organizations. Research advisory group members were involved in the study design, planning of data collection, analysis of data, and dissemination of results, which included a video, audio recorded, and written stories of the eight couples, and downloadable accessible data collection tools.

### Participants

Recruitment took place through UK-wide intellectual disability organizations, which acted as gatekeepers to distribute information to members. This was subsequently cascaded through social media and by contacting other UK intellectual disability and not-for-profit providers (non-governmental organizations). An overview of the inclusion and exclusion criteria for participants is provided in Table 1.

**Table 1.** Inclusion and Exclusion Criteria

Participant group	Inclusion criteria	Exclusion criteria
People with intellectual disability	Capacity to consent to take part. In a relationship lasting 6 months or more (or hasm been in a previous relationship for this length of time) with a person who has a diagnosis of dementia. If the partner has died, then the death must be more than 12 months before the participant is approached to take part in the study. This is to reduce potential distress during conversations about a sensitive topic. or Has dementia themselves and is in a relationship with a partner who has an intellectual disability. In either situation, both partners must be aware of the diagnosis of dementia and the relationship should be (or have previously been) for a minimum of 6 months.	Lacks capacity to consent to take part. In a relationship for less than 6 months. Has experienced bereavement of a partner during the previous 12 months.
Family members	Has/had a family member in a relationship where one partner has dementia (no time limit although the relationship should have been for a minimum of 6 months).	Their family member has been in a relationship for less than 6 months.
Social care staff	Has supported or currently supports a service user/client in a relationship where one partner has dementia (no time limit although the relationship should have been for a minimum of 6 months).	Couple have been in the relationship for less than 6 months.

When combined across cases, the 18 interviews conducted enabled us to develop an understanding of the experiences of eight couples with intellectual disability affected by dementia. Overall, we conducted life story interviews with five people with intellectual disability, including one who had dementia. Two participants were interviewed together as a couple at their request. All participants had capacity to take part in the study with written consent provided. This enabled us to include the perspectives of people with intellectual disability directly from couples 1, 2, 3, and 4 with family and social care staff supporting the couples also interviewed where possible.

Although we sought the views of people with intellectual disability as far as possible, we also included family and staff perspectives for a further four of the eight relationships (5, 6, 7, 8). See Table 2 for a breakdown of carer dyads and clarification of who was interviewed to build up each of the eight cases. In total, we conducted semistructured interviews with nine social care staff and four family members. The interviews took place throughout England and Scotland over a period of 8 months in 2022.

**Table 2.** Overview of Characteristics of Eight Couples With Intellectual Disability Pseudonyms Used for All Participants

Identifier	*Partner with dementia	Ages at time of interview in 2022	Relationship length	Type of dementia	Year of diagnosis	Overview of relationships at point of data collection	Did either partner have Down syndrome?	Participants providing data about each couple
Couple 1	Jimmy and *Ann	Jimmy 67 Ann 56 (in 2017 when she died)	20 years	Alzheimer’s disease	2011	Ann died in 2017 Couple lived together until Ann moved into a specialist group home supported by their intellectual disability service	Ann	Jimmy Ann’s sister Service Manager of care home Ann stayed during her last years of life. Known couple for 20 years
Couple 2	John and *Maggie	John 58 Maggie early 60s (when she died around 2010)	Around 20 years	Alzheimer’s disease	Around 2006	Maggie died in 2010 Couple lived together until this point	No	John
Couple 3	Laura and *David	Laura 57 David 67	More than 20 years	Vascular dementia	Around 2018	Midstage Couple live together supported by intellectual disability service	No	Laura Service Manager of supported living service Keyworker for Laura and David. Known couple for 14 years
Couple 4	Rose and *Tom	Rose 69 Tom 64	Almost 30 years	Dual diagnosis of vascular dementia and Alzheimer’s disease	2019	Early stage Couple live together	No	Rose Tom Current Service Manager. Has known couple for over 20 years
Couple 5	Ben and *Sarah	Ben 43 Sarah 52 (in 2020 when she died)	Around 13 years	Alzheimer’s disease (but mother not sure it was the right diagnosis)	Around 2017	Sarah died in 2020 Couple lived together until 2016 when Sarah moved closer to her mother	Ben and Sarah	Sarah’s mother Ben’s mother
Couple 6	Paul and *Mary	Paul 64 Mary 62	More than 20 years	Alzheimer’s disease Paul was also diagnosed with dementia at the end of the study	Around 2016	Advanced stage of dementia Couple lived together before both moved into a group setting supported by their intellectual disability service when Mary’s dementia progressed	Paul and Mary	Support worker for Paul and Mary during early stage of dementia. Known couple for over 15 years. Current Service Manager. Known couple for 6 years. Mary’s sister
Couple 7	Janet and *William	Janet 91 William 74	5 years (Couple were friends for over 10 years before their relationship started)	Specific type of dementia unknown to participants	Around 2018	Midstage Couple always lived separately. Janet now lives in a care home for older people	No	William’s local authority Care Manager. Has known William for over 15 years. Janet’s Support worker before her move into a nursing home. Has known Janet for over 20 years
Couple 8	Phoebe and *Adam	Phoebe 54 (in 2014 when Adam died, she was almost 60 when she died in 2019) Adam 56–57 (when he died in 2014)	More than 40 years	Vascular dementia Phoebe was also diagnosed with dementia a few years after Adam died	2012	Adam died in 2014 Couple lived together until this point	Phoebe and Adam	Phoebe and Adam’s Support Worker. Known couple for 7 years

As this is the first study to explore experiences of couples with intellectual disability affected by dementia, we included partners whose experiences were in the past as well as current, with the earliest timepoint of a dementia diagnosis in our sample being 2006 and the most recent in 2019. For some, this involved remembering events that had occurred many years earlier, while other participants were currently living with a partner who had dementia. We were aware of the benefit in collating information that spanned a wider time frame to maximize recruitment and to ensure we did not exclude valuable experiences. This allowed us to reflect on the impact of changing societal contexts for people with intellectual disability when developing individual life stories for each couple.

### Life Story Interviews With Couples

After discussion with the research advisory group, the decision was made to use visual supports with participants who had an intellectual disability to explore their relationship over time. Following Walmsley’s (1995) approach to life story research with people with intellectual disability, a life story map was created with signposts to the past, present, and future. We worked with an artist and the advisory group to create bespoke images that represented different stages in the relationship to frame the interview guide (see Supplementary Material 1). It was important that the images were used to guide the stories not lead them as we did not want to assume what might have been a positive or negative period in the relationship. They were designed in a way that did not make any assumptions about the ethnicity, age or gender of the couples, or even which partner had dementia. Some participants brought photo albums or had photographs on their mobile phones which supported the conversations.

### Semistructured Interviews With Family Members and Support Staff

Interviews with family and staff included questions about stages of the couple’s relationship, while also exploring the participants’ own role in providing support in the past and present, and their view on the couple’s future (see Supplementary Material 2). Length of interviews with people with intellectual disability ranged from 40 to 60 min and with staff and family members from 45 to 95 min. All were transcribed verbatim with names of participants and social care providers changed to ensure anonymity.

## Ethics

Ethical approval for the study was given by University of Stirling, NHS, Invasive or Clinical Research Committee (NICR 20/21 105) and NHS Health Research Authority Social Care Research Ethics Committee (20/IEC08/0042). Dementia can be a difficult subject to talk about and it was important to be prepared to support participants should anyone become distressed during or after interviews. Time was allocated before the interview to answer questions and ask what would help people to feel comfortable, for example, having someone with them or not wanting to talk about certain experiences. Time was spent with each participant at the end of each interview to reflect on the experience, finish with positive memories and, in relation to people with intellectual disability, to follow up with people’s support networks afterwards to ensure participants were well.

## Data Analysis

In line with Miles and Huberman (1994), analysis began with an in-depth exploration of the relationship of each couple. The importance of understanding the mutually dependent nature of relationships guided our analysis emphasizing that people with intellectual disability are active contributors in caring relationships. This also acknowledges that the lives of couples with intellectual disability often happen within wider webs of support, including support by social care workers and extended family members.

Accessible stories were created and discussed at three advisory group meetings, collaboratively identifying main themes for the eight different stories highlighting some differences in perspective between different participant groups. We then moved toward developing themes across couples and participants. Transcripts were checked and coded by one researcher initially for each participant group (people with intellectual disability, family members, and staff) before exploring connections and patterns across participants using NVivo12 software. Initial coding was guided by our predeveloped framework, which included codes about different stages in relationships and areas of interest identified from two systematic literature reviews conducted at the start of the study (Jacobs, Watchman, Wilkinson, & Hoyle, 2023; Jacobs, Watchman, Wilkinson, Hoyle, & McGenily, 2023). Deductive and inductive coding were combined to retain clear links to research questions and areas of interest, while also being open to unexpected findings (Fereday & Muir-Cochrane, 2006). For example, while information about how couples were involved in decision-making was identified as an area of interest beforehand, a connection between past experiences of marginalization and couple’s worries about the future was developed through inductive coding. Toward the end of the project, the advisory group met for a focused workshop to share and discuss themes across all interviews. An overview of the analytic process and theme development is provided in Table 3.

**Table 3.** Analytic Process

Stages	Analytic steps	Examples of coding
1. Precoding: Development of coding framework	Predeveloped codes based on interview guide and two literature reviews.	How couples met Life before dementia Dementia diagnosis Life after dementia Future planning Involvement in decision-making Couple’s understanding of dementia
2. Initial coding	Iterative coding of interview transcripts for each participant group separately. Development of predeveloped framework using NVivo 12. Development of new main and subcodes. The process was led by the one researcher, with another member of the team coding a subset of transcripts.	Life before dementia • Routines • Support networks Dementia diagnosis • Impact on couple • Process • Understanding of dementia Life with dementia • Partners as informal carers • Developing and changing understanding of dementia • Support • Routines and everyday life Past experiences of marginalization Impact of COVID-19
3. Development of themes across participants	Codes across participant groups were compared, arranged, and rearranged to identify connections. This involved moving back and forth between main codes, subcodes, initial research questions, and revisiting segments of the transcripts to develop themes. We moved away from different stages in relationships as the framework for codes toward themes that described shared experiences and differences across participants. Developing themes were discussed continuously within the team. Additionally, one researcher listened back to recordings of advisory group meetings that discussed the stories of each couple to ensure observations highlighted by advisers were reflected in the analysis. Toward the end of the project the advisory group came together for a focused workshop to share and discuss the developing themes.	Support and disruptions to relationships • Changes in support over time • Impact of COVID-19 • Changes in relationships, routines, and support to maintain relationships • Staying together and moves Impact and understanding of dementia diagnosis • Receiving the dementia diagnosis • Impact on couple • Support to make sense of dementia • Changing understanding of dementia over time • Lack of accessible resources Partners as informal carers • Partner taking on caring roles • Support for partner without dementia Involvement • Involvement in future planning • Involvement in dementia diagnosis • Worries about move to care home linked to fears about institutional care • Past experiences of marginalization and not being listened to • Staff not fully informed about intellectual disability and dementia

## Limitations

Although multiple perspectives were sought to develop cases, this remains a small sample size, with participants all White and UK-based. However, such a small size is not unusual in qualitative research, and we recognize that this group is typically less often included in research. For this reason, although narratives from four of the eight couples included the voice and the direct experience of people with an intellectual disability, a further four narratives did not include the couple directly. Whilst this enabled us to develop understanding of professional and family experiences, the perspectives of the couples with intellectual disability themselves may have been different. All partners received support from an intellectual disability service provider, which is not representative of the living situation of couples who may have less supportive networks. Despite a gender balance in participants with intellectual disability, all family members and professionals were female.

## Results

This section begins with an overview of the characteristics of couples, before discussing experiences in relation to: (1) support to maintain, and disruptions to, relationships; (2) impact and making sense of the dementia diagnosis; (3) partners as informal carers; and (4) involvement in decision-making. Couples 1, 2, 3, and 4 were cases that directly included one or both partners with intellectual disability (and dementia in one case) in addition to family and support staff. Couples 5, 6, 7, and 8 represent perspectives of family and support staff only.

### Couple Characteristics

The eight couples in our study had been together for a combined total of 170 years highlighting longevity in relationships with most lasting for more than 20 years. Couples were at different stages in relation to the progression of dementia when data were collected. For one couple, the partner with dementia had, to that point, experienced few symptoms and continued to be largely independent (4). Two partners with dementia had experienced an increase in support needs (3, 7), including decrease in verbal communication and appearing more forgetful and anxious in busy or unknown environments. One partner was in the advanced stage of dementia with a marked decline in verbal communication and the need for mobility aids (6). In three of the cases, the partner with dementia had died (1, 2, 5) and in one case both partners had died (8).

Three partners with dementia who had died (1, 5, 8) had Down syndrome and the progression of their dementia appeared to have been more rapid from diagnosis to death at 2, 3, and 6 years. These three partners experienced a decline in living skills and reduced verbal interaction with an associated need for increased support earlier than reported in the other couples.

#### Support for, and disruptions to, relationships

Being in a relationship was reported to have a positive impact on the lives of all couples, providing intimacy and belonging that continued as dementia advanced. Access to consistent support networks varied between couples; however, at the time of the dementia diagnosis all couples were receiving support from intellectual disability services to live independently. Four couples had close family involvement (1, 2, 5, 6), two had intermittent contact (3, 8), and for two couples an absence of family involvement was reported (4, 7). Six couples had been supported by the same intellectual disability provider for many years with consistent support from some members of staff (1, 3, 4, 6, 7, 8). Early signs of dementia could be subtle, and it helped when staff and family members saw people regularly enough to notice changes and to start the process of involving health professionals. High turnover of social care staff leading to a lack of intellectual disability and dementia knowledge within support organisations was described as a barrier to noticing changes and monitoring dementia progression. In two cases, the partner without dementia was described as alerting others to early changes.

They were and still are a very routine couple so he would alert staff to say she is not home yet or she got on the wrong bus. (Staff, couple 6)

As dementia progressed, interactions between partners were described as decreasing, with more conflict for some. However, despite changes to relationships there continued to be love and care between partners.

We used to have a nice time together, even those times, like she used to hold my hand, I used to hold her hand and we used to cuddle and kiss and she’d go “sorry.” I’d say, “Oh don’t worry about it, it’s all right, it wasn’t your fault.” And then we’d have a cuddle and then we’d be all right together. (Partner without dementia, couple 2)

Being supported to maintain routines, even a new routine, helped couples to retain an element of control among the changes. This included family members stepping in to support annual trips and holidays. Staff and family members described how looking at photo albums, playing favorite songs of the couple, preparing favorite food, and reminiscing with the couple helped to support positive interactions, even at the advanced stage of dementia.

I set up the headphones for Ann and then gave Jimmy his, and I switched it on, and it was one of those moments that kind of will stay with me for life. Because as soon as I put the music on, they were like in a bubble, and she was gazing at him, and the two of them were smiling at each other, and they were singing along to the songs. (Service Manager, couple 1)

Two couples were supported by their intellectual disability service, alongside support from health professionals and palliative care teams, to continue living together in their home (2, 8). Four couples experienced accommodation changes. Disrupted sleep and its impact on the partner without dementia were identified as a main concern for couple 6 who moved together to a 24-hr supported group home along with others who had an intellectual disability. In couple 1, the partner with dementia moved into a specialist dementia care provision managed by their intellectual disability service as her dementia advanced. Her partner stayed in their house and visited daily. In couple 5, the partner with dementia moved from their shared flat to live with family due to their concerns about the intellectual disability service being able to support her changing health needs. As her mobility declined, she later moved into a residential care home for older people. In couple 7, it was the partner without dementia who moved into a residential care home for older people after a fall. Moves to care homes by partners in couples 5 and 7 resulted in disruptions to relationships. In both cases, support staff were able to maintain contact with the resident themselves providing invaluable information to care home staff about preferred routines and communication. They were also able to support visits by the partner for as long as possible. For both couples, the COVID-19 pandemic resulted in a prolonged physical separation as partners were unable to visit. This was described by staff and family members as a very distressing experience for both couples. The COVID-19 pandemic also disrupted existing support networks for the three couples who lived together (3, 4, 6) as family members were unable to visit and social groups and employment stopped. Couples felt more isolated and staff and family members believed that the sudden halt to routines and activities had contributed to advancing dementia symptoms and increased anxiety.

The importance of ongoing training ito understand how dementia may affect people with intellectual disability was identified for staff teams and families. Multidisciplinary collaboration and sharing information between intellectual disability staff, health professionals, palliative care teams, and family members were described as essential. Health professionals provided important information to understand and manage new symptoms, including the use of medication and equipment to allow people to stay in their home. Social care staff and family members who knew couples well were integral to offering emotional support, including supporting couples to manage hospital visits.

#### Impact, and making sense, of the dementia diagnosis

There were differences in experiences of receiving a timely dementia diagnosis. For three couples (1, 5, 7) staff and family members remembered that the process took several years. At early stages, it was difficult to discern if symptoms could be an extension of existing support needs. Additionally, four partners with dementia (1, 5, 7, 8) experienced concurrent health problems, including visual impairment, mobility issues, skin infections, irritable bowel syndrome, and a heart condition. These exacerbated support needs and potentially delayed the assessment process.

Her hip was giving her pain, she was limping. Her GP said to me there’s no point in us trying to do any sort of assessment with all this other stuff clouding the issue. (Family member, couple 5)

Receiving the dementia diagnosis evoked strong emotions from four of the couples (2, 3, 4, 6) who verbalized feelings of loss, distress, and anxiety. Three partners expressed how it was frightening to anticipate changes without knowing if or when they would occur (2, 3, 4). One partner with dementia (4), talked about his concern that he might start to be aggressive toward his wife, despite an absence of any occurrence of aggression between the couple to that point.

What if my behaviour to my wife changes, how will we cope? (Partner with dementia, couple 4)

Additionally, three partners without dementia (1, 2, 3) and one partner with dementia (4) said they were struggling with the terminal nature of the illness. Each spoke about the importance of knowing what could be done to help their partner or themselves in the present.

When the people told me about you can’t do nothing about dementia, I used to argue with some of the staff. Well not really argue but disagree with them. I used to say to them, no, she is going to get better. (Partner without dementia, couple 2)

The dementia diagnosis was shared with all couples although there were differences in relation to the exact words that were used and how much detail was shared. The terms dementia or Alzheimer’s disease were not used at the early stage for two couples (1, 5). Instead, staff and family members focused on explaining the changes people were experiencing at that time. For five couples (1, 3, 4, 5, 6), partners and staff members valued the use of intellectual disability and dementia-specific resources, such as social stories, accessible information or videos, which helped to facilitate conversations.

He had the cards from Jenny’s Diary (Watchman et al., 2015), and it has the pictures of people with dementia. [His partner] watched the Supporting Derek video (Watchman et al., 2018). She sat down with a member of staff, so that gave her a lot of understanding of what dementia was. (Service Manager, couple 3)

Although conversations about dementia were hard, could increase people’s worries and at times lead to confusion, not talking about dementia further increased anxiety. All five partners explained how they had learned more about dementia through television representations or knowing other people with dementia. Regular conversations with staff and family members helped some couples to be reassured and to understand that dementia might be different for their partner or themselves. All five partners said that they felt more comfortable talking to some individuals rather than others and wanted choice over this. For two partners without dementia and one partner with dementia, services had arranged counseling sessions, which were described as very positive, although it was challenging to find counselors with experience of working with people with intellectual disability.

Learning from the cases where a partner had died highlighted that information about death was easier for partners without dementia to understand and accept during the advanced stage of dementia when decline was visible.

In the very, very final stages of her life, he understood she would pass, but right up until then, he was always waiting for her to get better. (Family member, couple 1)

#### Partners as informal carers

Partners described how dynamics in the relationship changed as dementia progressed. The individual without dementia started to make more decisions on behalf of the couple. Accepting help from their partner could be difficult for people with dementia and two partners said that it could lead to conflict.

I’m trying to help him and he wants to be independent. I say, “Want me to help?”, but he says no, he wants to do it properly all on his own. (Partner without dementia, couple 3)

Some took on new caring roles and helped their partner with dementia in their everyday lives. In three couples, this included practical support such maintaining self-help skills, assisting with household tasks, or calling for medical help.

When she took a seizure, you felt it coming. Then I had to phone for the doctor, the doctor had to give her an injection. (Partner without dementia, couple 1)

Partners helped with personal care, taking a bath or shower, assisting with the use of hoists, or feeding in the advanced stage of dementia.

I’d put her clothes on for her, help her and put her trousers on, her tops on, her coat on for her. And then I used to feed her sometimes as well. Sometimes the staff used to feed her and sometimes I used to feed her. I had to put the food on the spoon or fork and feed her, help her. (Partner without dementia, couple 2)

Partners talked about how their caring role could be scary and worrying, but also expressed how they wanted to be involved. Three discussed how it had been difficult to respond to verbal and physical aggression by their partner (1, 2, 3). Talking to staff and realizing that this was due to frustration, and that changes were as a result of dementia, helped people. Identifying ways to respond such as leaving the room for a while, calling a member of staff, or engaging in calming activities were described as successful strategies to maintain a positive relationship between partners.

The staff saying, she can’t help it when she’s saying that. She still loves you. [That was helpful]. (Partner without dementia, couple 1)

The family members who took part were all female, two sisters and two mothers. They provided early memories of the childhood of people with intellectual disability and early experiences of stigma and exclusion. Three family members (1, 5, 6) described how the relationship had challenged their own and others’ views and assumptions, how they and other relatives had been surprised by the wish of couples to be together or had not expected that their family member could be as committed and caring. Interviews illustrated how partners became part of extended families and how relationships with parents or sibling in-laws continued after the death of the person with dementia.

#### Involvement in decision-making

One couple (4) had been supported to speak with a lawyer to plan ahead and manage legal affairs. A further couple (6) had spoken about and written down their preferences about where they would like to live should their health deteriorate and what kind of funerals they would like. Staff members stressed how important it was that planning involved both partners separately as wishes and preferences might not always be the same. For example, one partner said in her interview that she was not sure if she would want to continue living with her partner as his dementia progressed.

Although interviews with all participants included positive examples of services and family supporting relationships and helping couples to think about and plan for the future, there were also examples of decisions being made without talking to both partners. This included staff making changes to one couple’s house to make it more accessible without involving them, which led to conflict. For three of the couples, visits between partners or visits to family members were at times disrupted due to staff changes and/or staff shortages. It was apparent that couples relied on staff and family members to support relationships, and this was particularly apparent for the three couples who had been physically separated through moves to a care home.

Two people with intellectual disability spoke about a fear of being separated from their partner in connection to past experiences of not being involved in making decisions. All couples, to varying degrees, had previously needed to ask for permission to be in a relationship. Past experiences of shared living and institutional care were revisited when talking about planning ahead with concerns raised about a move to a care home and the associated perception of a large congregate setting. It became apparent that the couple’s life stories were important historic artifacts and outputs in themselves. They told of relationships over decades as the couples lived through changing approaches toward intellectual disability in the UK from institutional living through care in the community to independent living. One couple had met in the 1970s in one of the biggest intellectual disability hospitals in Scotland before moving to their own home in the local community when the institution was closed in the late 1980s. Other couples started their relationships while living in group homes or while sharing flats with other people. Having their own home together later in their relationship gave couples privacy and a sense of belonging.

[When we moved into our house] we didn’t have any people barging in or things like that. The staff came over every day at certain times but never stayed. They didn’t do sleepovers or anything like that. The house was ours. I don’t want to move back to one of them places (institution/long stay hospital). (Partner with dementia, couple 4)

## Discussion and Implications

This is the first study to explore the experiences of couples with intellectual disability affected by dementia. By looking at a range of perceptions, we were able to explore lives of the couples themselves in addition to identifying support needs. Couples drew on support from staff and family they knew well and trusted with partners providing emotional support for each other. Moments of physical intimacy and closeness continued to be important to maintain a sense of belonging and being as a couple.

Although a direct comparison is not possible, our findings show similarities in experiences to couples without intellectual disability affected by dementia. In both contexts, couples spoke about feelings of loss and fears about the future as their relationship changed, with regular respite and emotional support identified as important for partners without dementia (Egilstrod et al., 2019). However, our study also highlights differences and additional challenges that couples with intellectual disability faced. The care and support needs of couples without intellectual disability affected by dementia often remain located within the private lives of the couples, with an absence of social care support in the early and midstages of dementia (Kerpershoek et al., 2019). This is a significant difference for our couples, who had existing and immediate access to staff in the early stage of dementia. None of our couples had children, and there were differences in availability of support from family members, again reflecting a greater reliance on social care services rather than being able to draw on informal support networks (Power & Bartlett, 2019). Only one of the partners discussed that they had talked to staff and health professionals about having children but had decided against this, further highlighting how for couples with intellectual disability private conversations and decisions are managed or at least often facilitated by staff or family members (McCarthy et al., 2021; Neuman, 2020). Longstanding relationships between staff and couples were noticeable. Social care staff interviewed had known the couples for many years, two for over 20 years, four for over 15 years, one for 7 years, and one for 6 years. This meant that life stories were known, which have been identified as helping support people to maintain relationships and promote personhood in research with couples without intellectual disability affected by dementia (Smebye & Kirkevold, 2013). Conversely, staff support could at times also work as a barrier and negate the involvement of both partners in decision-making, which appeared to be linked to views of people with intellectual disability as vulnerable or incapable (McCarthy et al., 2021). This dependence on social care services to facilitate work and social lives led to increased isolation during the COVID-19 pandemic for some of the couples. Similar to studies about people with dementia without intellectual disability (Hanna et al., 2022) it was identified that COVID-19 had appeared to speed up a decline in skills for partners with dementia.

Partners spoke of their concerns about moves to care homes in light of past experiences of not being listened to, institutional living, and some experiences of group homes. Those that supported couples with intellectual disability affected by dementia did not always understand past and prevailing experiences of marginalization and stigma, thus minimizing their ability to understand responses to experiences of loss and anxieties about the future. Neuman (2020) identified that living in one’s own home is of accentuated significance for older couples with intellectual disability who have experienced institutionalization and shared living when they were younger. Our study has contributed to a wider understanding in practice of how stigma associated with dementia continues to impact negatively on people living with the dual diagnosis of intellectual disability and dementia. It demonstrates the importance of promoting a working culture that recognizes individual experiences and of involving people with dementia in decision-making. This was reflected in the evidence review informing the new Dementia Strategy for Scotland (Huang et al. 2023), which referred to our research when acknowledging the practical guidance required to meet the complex needs of people with both intellectual disability and dementia.

The impact of the dementia diagnosis and need for emotional support were evident throughout the research. Rather than asking for a definition of dementia, couples wanted to know what dementia would specifically mean for themselves and their partner. Three people with intellectual disability received counseling, challenging views that counseling is not accessible for people with intellectual disability (Taylor, 2010), or indeed for people with dementia generally (Griffiths et al., 2020). In the few studies that exist on people with intellectual disability as informal carers, the focus has been on relationships between people with intellectual disability and their aging parents (Truesdale et al., 2021). Similar to these studies, our data show how people with intellectual disability helped their partner by taking over domestic tasks, helping with personal care, support with eating, drinking, and medication, as well as providing emotional support and reassurance. Although partners needed support with their caring role, they also wanted to care for their spouse and be involved in decisions about their care (Truesdale et al., 2021).

Couples in this study had all been informed of the dementia diagnosis, which does not always happen for people with intellectual disability (Sheth, 2019). Consequently, we were able to explore people’s experiences more directly by talking specifically about dementia and drawing on our accessible interview process. Our study challenges views that people with intellectual disability do not understand or cannot talk about dementia, but we identified that additional supports were required to make sense of their dementia experiences. Consistent with the model developed by Tuffrey-Wijne and Watchman (2015), which provides a framework for conversations about dementia with people with intellectual disability, we found that understanding was a process that required the revisiting of information, often on an “as needed” basis or at that specific point in time.

## Summary

Our study has shown that people with intellectual disabilities affected by dementia can successfully maintain intimate relationships. Their experiences suggest that support of family members and professionals with knowledge and information of how dementia can affect people with intellectual disabilities is essential to overcome societal and other barriers that people with intellectual disabilities often face in order to give expression to their human rights. The importance of multiagency support and interdisciplinary intellectual disability and dementia training alongside family members who knew the couples well were highlighted as crucial in sustaining relationships as dementia progressed. A diagnosis of dementia presents unique challenges to couples in part due to past experiences. This includes having to fight to be allowed a relationship, to live together and to be listened to. Changing demographics, including increased life expectancy for people with intellectual disabilities mean an increased number living with a partner with dementia or receiving a diagnosis themselves. It is therefore important for services to understand the additional complexities, and to ensure that people with intellectual disabilities receive appropriate support so that, like everyone, they live as well as possible with dementia.

## Supplementary Material

gnae030_suppl_Supplementary_Materials

## Data Availability

Data can be accessed for replication purposes at DataSTORRE: Stirling Online Repository for Research Data http://hdl.handle.net/11667/212. This study was not preregistered.
