# Supplementary material for: “How will we cope?” Couples With Intellectual Disability Where One Partner Has a Diagnosis of Dementia
Source: Gerontologist. 2024 Mar 20;64(6):gnae030. doi: 10.1093/geront/gnae030 (PMC11127106; doi:10.1093/geront/gnae030)
Supplement: gnae030_suppl_Supplementary_Materials [file gnae030_suppl_supplementary_materials.docx]

**Supplementary Material**

**Supplementary Material 1**

Roadmap with example of commissioned images to support life story interviews


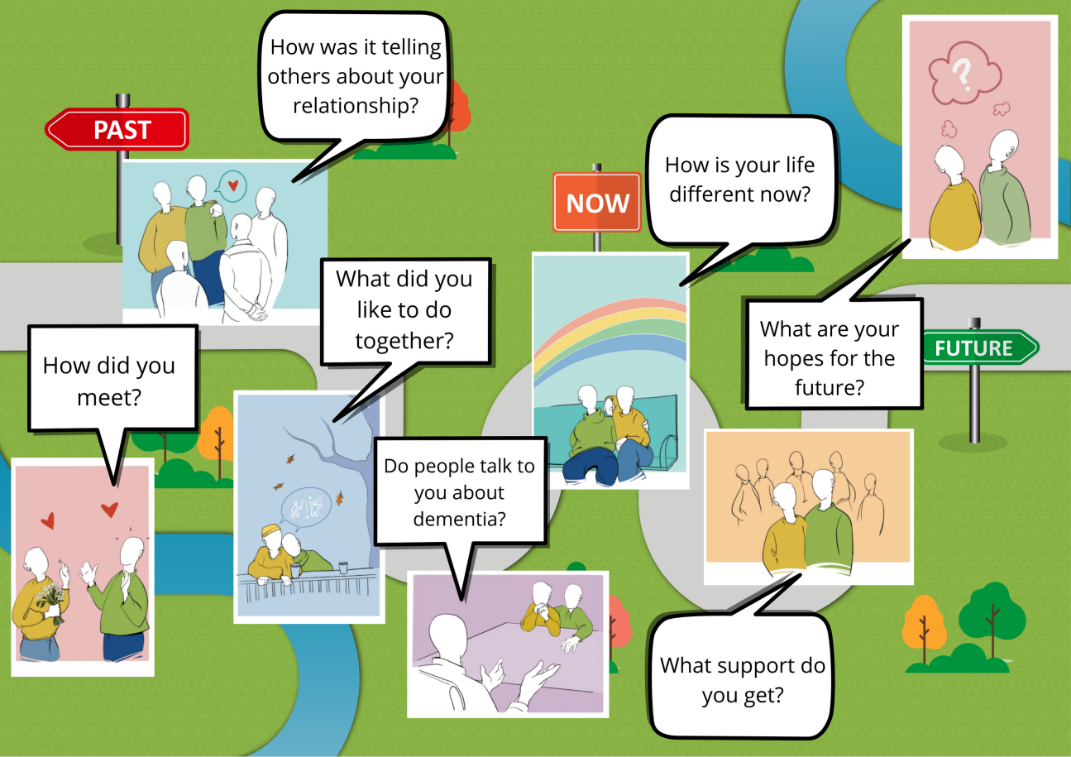


**Supplementary Material 2: Interview Schedules**

**Interview schedule - couples**

| Relationship stage | Questions we will ask people |
| --- | --- |
| First meeting | How did you meet?  Was it love at first sight?  What did you like about your partner when you first met?  How long have you been together? |
| Telling others | What did your family or workers think about your relationship?  How was it telling others about your relationship?  (What did they think about you wanting to get married or move in together?) |
| Life as a couple in the past | What did you do before you met (name of person)?  How did life change when you became a couple?  What activities did you like to do together when you first met?  What did your week look like?  (Add questions about any known key-events such as marriage, holidays or moving together) |
| Dementia and changes | I would now like to talk to you about the time when you and others noticed that your partner has an illness called dementia. Is that ok?  Do people talk to you about dementia?  Dementia means that your partner’s brain is not working so well anymore. It is not their fault.  Are there things that your partner finds more difficult because of dementia? |
| Support of others | Who are important people in your life?  How do they help you?  How often do you see them?  Has the support you get changed because of dementia?  Are there new people in your life now because of dementia?  Do people support you with things like managing money or making future plans? (lawyer etc, power of attorney) |
| Life as a couple now | How does your life as a couple look like now?  What do you do together now?  Is life different for you now as a couple, than it was before? (going to work/day centre/the shops...)  What is working well?  Is there anything that is difficult?  What is helping you when things are difficult? |
| Life next | What are your hopes for the future (what comes next)?  If you had a magic wand, what would you change? |
| Other questions | Is there anything else you can think about that you would like to tell me?  Do you have any questions you would like to ask me? |
| Ending the interview | What is your happiest memory of being with your partner?  What is your favourite thing about them?  How was it for you to do the interview?  Is there anything I could have done better?  What are your plans for the rest of the day/weekend? |

**Interview schedule - staff and family members**

1. Can you tell me about (person’s name or couple)?

- How long have you known (person’s name and/or couple)?
- Can you tell me about your role in the person’s life (couple’s life)?

2. How did (names of couple) meet?

- How long have they been together?
- How did you and others initially feel about the relationship? (How do you feel now?)
- What has the journey as a couple been like for them?
- How did their life look like before the dementia diagnosis? (everyday-life, ups and downs, supporting each other, support provided to/received by couple and each partner)
- Did you provide support to the couple (or one partner) before the dementia diagnosis (and if yes what did this look like)?

3. When did people first suspect the possibility of dementia for (person’s name)?

- What changes did you and others notice? How did the initial changes affect the couple?
- How was the process of getting the diagnosis of dementia? What type of dementia was diagnosed?
- What is each partner’s understanding of the dementia diagnosis?
- How did the diagnosis affect the couple? How did it affect each partner?

4. Changes and support needs

- How has dementia progressed since the diagnosis?
- What support is provided to the couple and each partner? (within the organisation /family and external supports) Did the support change?
- Who are important people in the life of the couple and each partner?
- Which professionals are involved? (any constant from before or are they new?)
- What is the legal situation of each partner? (power of attorney, guardianship)
- Has your role in the couple’s or person’s life changed? Has dementia affected other people in the life of the couple or each partner?

5. How is the life of the couple now?

- Is it different to before? (how have roles, tasks and responsibilities changed?)
- What is working well? What is difficult? What do you think is needed to overcome those difficulties?

6. How do you feel about the future of (couple)?

- Do the couple or each partner talk about the future? Have you talked to the couple/partner about the future (as part of care plan)?
- What do you think would help to maintain the relationship?
- What, if any, changes are anticipated in the future and in what timeframe is the change(s) expected? (Maybe accommodation, social activities, level of support, night time support, end of life/palliative care – if changes are identified we can explore why this may be, what the anticipated impact will be on each partner and if either or both partners are/will be involved in planning for change – and how)

7. Do you have any other thoughts or would like to mention something I have not asked about?

8. Look at road map together and identify key-stages/events
